# Mobile Apps to Support Healthy Family Food Provision: Systematic Assessment of Popular, Commercially Available Apps

**DOI:** 10.2196/11867

**Published:** 2018-12-21

**Authors:** Chelsea E Mauch, Thomas P Wycherley, Rachel A Laws, Brittany J Johnson, Lucinda K Bell, Rebecca K Golley

**Affiliations:** 1 Nutrition and Dietetics College of Nursing and Health Sciences Flinders University Bedford Park Australia; 2 School of Pharmacy and Medical Sciences Division of Health Sciences University of South Australia Adelaide Australia; 3 School of Health Sciences Division of Health Sciences University of South Australia Adelaide Australia; 4 Institute for Physical Activity and Nutrition School of Exercise and Nutrition Science Deakin University Geelong Australia

**Keywords:** diet, nutrition, family, mobile applications, behavior modification

## Abstract

**Background:**

Modern families are facing conflicting demands on their time and resources, which may be at the detriment of child and family diet quality. Innovative nutrition interventions providing parents with behavioral support for the provision of healthy food could alleviate this issue. Mobile apps have the potential to deliver such interventions by providing practical behavioral support remotely, interactively, and in context.

**Objective:**

This review aimed to identify and assess popular, commercially available food- and nutrition-related mobile apps that offer support for the provision of healthy family food by (1) describing app scope and characteristics, (2) assessing app quality, and (3) conducting a behavioral analysis of app content and features.

**Methods:**

Searches in the Google Play Store and Apple App Store between August 2017 and November 2017 identified apps addressing the food provision process. Apps were included if they were applicable to parents or families, written in English, and with a user rating of ≥4 stars. Weight loss and diet monitoring apps and subscription apps with no free versions were excluded. App quality was assessed using the Mobile App Rating Scale (4 domains: engagement, functionality, aesthetics, and information). App content and features were extracted and behavior change techniques (BCTs) identified.

**Results:**

Of the 2881 apps screened, 1.77% (51/2881) were included for assessment, comprising 23 recipe and recipe manager apps, 12 meal planning apps, 10 shopping list apps, 4 family organizers, and 2 food choice apps. Half (n=26) of the apps functioned primarily through user data input. Food choice and family organizer apps scored highest for app quality (mean 3.5 [SD 0.6] out of 5), whereas most apps scored well for functionality and poorly for engagement. Common app features with the potential to support healthy food provision included meal planners (n=26), shopping lists (n=44), and the ability to share app content (n=48). Behavioral support features mapped to relatively few BCTs (mean 3.9 [SD 1.9] per app), with *Adding objects to the environment* present in all apps, and 65% (33/51) including *Instruction on how to perform the behavior*.

**Conclusions:**

Recipe and recipe manager apps, meal planning apps, and family organizers with integrated meal planning and shopping lists scored well for functionality and incorporated behavioral support features that could be used to address barriers to healthy food provision, although features were focused on planning behaviors. Future apps should combine a range of features such as meal planners, shopping lists, simple recipes, reminders and prompts, and food ordering to reduce the burden of the food provision pathway and incorporate a range of BCTs to maximize behavior change potential. Researchers and developers should consider features and content that improve the engagement quality of such apps.

## Introduction

### Background

Excessive consumption of energy-dense, nutrient-poor foods is a key cause of poor diet quality [1-4] and is contributing to the high prevalence of overweight and obesity globally [5-7]. In Australia, these foods are contributing 30% to 40% of the total daily energy intake of children and adolescents [2]. Similar figures have been reported in the United States and Canada, with children and adolescents consuming at least one-third of their daily energy intake in the form of energy-dense, nutrient-poor foods [4,8]. The increasing reliance on these generally highly processed foods may be in part because of the conflicting demands that the modern lifestyle places on the resources available for family food provision [9].

Food provision, encompassing the planning, purchasing, and preparation of food, requires significant time and both mental (eg, food preparation knowledge and planning skills) and physical (eg, food preparation facilities) resource [10-12]. The use of food coping strategies (such as meal planning, shopping list writing, use of convenience ingredients or preprepared meals, and seeking support) can enable families to overcome resource-related barriers to food provision (including time or income scarcity). Although some strategies, such as the purchase of fast or convenience food, occur at the detriment of diet quality [13-17], other strategies, such as meal planning and shopping list use, have been associated with healthier food preparation [13]. Nutrition interventions supporting the use of healthy food coping strategies are warranted and in fact desired by parents [18]. However, interventions supporting parents to improve their children’s dietary intake are primarily focused on education rather than skill development and are of moderate effectiveness [19]. Addressing resource-related food provision barriers and supporting the adoption of healthy food coping behaviors may enhance the effectiveness of interventions to improve child and family diet quality [11,12,20].

Health interventions delivered by mobile apps have the potential to address resource-related barriers to healthy food provision by offering practical behavioral support, remotely, interactively, and in context [21]. The unique placement of mobile phones within our daily lives, along with technological advancements such as global positioning system, machine learning, and data tracking, means that apps are positioned to deliver ecological momentary interventions [21,22]. Although the initial time and monetary outlay for app development can be substantial, they are highly scalable, and with mobile phone ownership nearing saturation, they have the potential to reach a diverse population [23,24]. Furthermore, interventions can be personalized based on user input, which may improve user engagement and intervention fidelity [22,23]. The current popularity of health and nutrition-related apps in both the general public and in research, along with the opportunities that the technology provides, makes it an important platform to explore for future family nutrition interventions [23,25].

### Reviews of Mobile Health Interventions and Commercially Available Apps

Reviews of nutrition-related mobile health interventions have examined their effectiveness in relation to behavioral and weight-related outcomes [26-29]. A meta-analysis of 12 diet and physical activity–focused app studies found that delivery of an intervention via a mobile app significantly reduced weight compared with controls (−1.04 kg, 95% CI −1.80 to −0.27 kg) [27]. Similarly, a systematic review found moderate evidence that diet and physical activity apps lead to improvements in health-related behaviors and outcomes (19 of 27 apps) [29]. However, these reviews have generally focused on apps for weight loss or diet monitoring, with limited relevance to family food provision [26-29].

A recent scoping review identified studies describing apps relevant to families, although the focus was primarily on apps supporting parent food practices (ie, responding to vegetable refusal and food portions) and monitoring of family members snack intake [30]. The same review identified a small subset (19%, 9/47) of mainly app development studies describing food access and food purchasing apps [30]. These apps were found to utilize environmental support features such as recipe suggestions and augmented reality tagging of products in the supermarket aisle [30]. Therefore, although there is evidence of the development of apps providing behavioral support for aspects of food provision, there is a paucity of published research exploring the use of apps for families that consider a range of food provision processes. To understand the potential role of apps in addressing a range of food provision processes, it is crucial to look toward existing, commercially available apps to support innovation in future research studies [23].

Reviews of apps in the commercial space have assessed app features and quality as well as identified the behavior change technique (BCT) content of nutrition, physical activity, and weight management apps targeting adults [31-33] and children [34,35]. These reviews found that there remains a need to enhance app quality and utilize behavior change theory in app development as important precursors to app effectiveness [31,33,34]. The focus of these apps on diet and weight-related outcomes (such as calorie counting and weight monitoring), rather than the behaviors leading to healthy dietary intake and weight, may limit their behavior change potential [33]. Similar to reviews of published app studies, commercial apps pertaining to food provision in a family context have yet to be explored. To ensure that current technological and behavior change potential in this area is fully understood, and to understand gaps in the commercial space, a review of existing, commercial apps addressing family food provision is required.

### Objectives

Thus, the purpose of this review was to identify and assess popular, commercially available food and nutrition-related mobile apps that have the potential to offer behavioral support for the provision of healthy family food. Specifically, the objectives of this systematic assessment were to describe app scope and characteristics, assess app quality, and conduct a behavioral analysis of app content and features.

## Methods

### Search Strategy

Systematic searches were conducted in the Google Play Store and Apple App Store between August 2017 and November 2017. The search strategy was modeled on prior systematic assessments in similar fields of research [31,32,34,36]. Google Play searches were conducted on a personal computer in a Google Chrome Web browser without Google account log-in. App Store searches were performed using the app on an iPad, as the store does not include a search function when used on a personal computer [32]. Search terms relating to the food provision process were selected, and pilot searches in both stores resulted in the following primary terms being used to identify apps for inclusion:

- WHO: child, children, toddler, kid, kids, preschooler, family, families, and parent
- WHAT: nutrition, food, meal, menu, recipe, recipes, and diet
- HOW: planning, planner, shopping, supermarket, grocery, budget, cook, cooking, prep, and preparation

Terms were combined into groups reflecting the various stages of the food provision process, including meal planning; food budgeting; nutrition, food, and cooking knowledge; food purchasing; and meal preparation. Combinations of 2 to 3 words were then generated for each group (eg, meal planner and child meal plan), and the first combination from a group was entered, with the first 50 results being checked by title and description against the inclusion and exclusion criteria. This was repeated for subsequent search terms from that group until a term returned no new apps that met the inclusion criteria. The search was then deemed saturated for that group and the next group of search terms applied.

### App Selection

Apps were included if they were applicable to parents with children, written in the English language, and had a user rating of at least four stars in the Google Play Store (to ensure that only popular, functional apps were reviewed) [31]. This limit was not applicable in the App Store as most apps had insufficient reviews to be given a star rating. All free, paid, and freemium apps were included, except where the app was subscription only with no freemium version. The following app types were excluded: (1) weight loss, diet monitoring, and calorie counter apps; (2) generic apps with only 1 food-related component (ie, personal organizers with a shopping list); (3) infant food and feeding apps; (4) apps focused on child feeding practices, electronic books, or magazines; and (5) recipe apps focused on unhealthy food (ie, cakes) or 1 key ingredient or cuisine. Apps were also excluded if their use was contingent upon involvement in a research study or a face-to-face component. The initial screen using these criteria was conducted using the app name, description, and screenshots of the app found within the stores. Approximately 10% of the screened apps (selected randomly, using the random number function within Microsoft Excel 2016) were checked by a second reviewer for correct inclusion and exclusion. Agreement was 93.7% (256/273), with discrepancies discussed and consensus reached [36].

Due to large numbers of similar and generic apps (eg, basic shopping list apps), a second and third screen was undertaken with additional exclusion criteria. At the second screen, apps with only 1 food-related component (ie, recipes only), less than 20 reviews in the Google Play store [34], and duplicates between stores were excluded. Apps were then grouped according to their primary purpose as described in the Google Play Store or App Store, and a third screen applied to ensure that the final sample provided good representation of the features available in such apps. Using the app description in the Google Play Store and App Store, apps were included if they had at least one unique feature not yet described in another app from that group of apps, or features in a unique combination.

### Data Extraction and Assessment

Once all eligible apps were identified, an Apple iPad Mini Version 4 (Model A1550) and Lenovo Tab3 7 Essential (Model TB3-710F) were used to download apps for assessment. Where apps were *freemium* (ie, available for free but with some features only accessible with payment), the paid version was purchased, except where subscription was required. These apps were downloaded and assessed in the free version. Apps were used for a minimum of 10 min before any data extraction or assessment took place [37]. Reviewers used individual apps for a period of time (generally on a number of occasions) that was sufficient to familiarize themselves with the apps features and functionality. The time spent using apps varied because of the significant heterogeneity of the included apps. Data extraction was checked, and apps were assessed independently by a second reviewer in a random sample of 22% (11/51).

#### App Characteristics

App information including app and developer name, operating system availability, version, affiliations, cost structure, user rating and number of downloads (where available), and app scope (ie, target audience and behavior) was extracted into a purpose-designed Microsoft Excel 2016 spreadsheet. The primary direction of data into or out of the app was determined and described as input, output, or both. App content such as information, videos, images, and recipes were defined as *output*, whereas features requiring user input, such as entering items into shopping lists or meal planners, were defined as *input*.

#### App Quality

App quality was assessed using the Mobile App Rating Scale (MARS), an objective and reliable measure of the quality of health-related apps [37]. The domains assessed by the MARS tool include engagement, functionality, aesthetics, and information [37]. An optional domain regarding subjective app quality was not included in this study. Apps were rated between 1 and 5 for each of the criteria, with 4 mean domain scores and an overall mean score across all 4 domains being indicative of app quality (a score of 5 indicating the best performing apps). Both reviewers viewed a Web-based training video before app assessment [38]. Inter-rater reliability of the overall MARS score was tested on the sample of double-assessed apps using the two-way random effects intraclass correlation coefficient (ICC) [39]. The resulting ICC value of .74 indicated good inter-rater reliability [40].

#### App Content and Features

Data regarding app content and features were sorted into 2 distinct categories: (1) “Behavioral support content and features” and (2) “Technical features.” “Behavioral support content and features” were those that may enable the performance of a behavior relating to the provision of healthy family food. “Technical features” did not offer behavioral support but were important to the overall functioning of the app. App content was then assessed for the presence of BCTs against the BCT taxonomy version 1 (BCTTv1) [41]. Both reviewers underwent Web-based training before coding [42]. The agreement between reviewers regarding the presence of BCTs was tested in the 11 double-assessed apps using kappa and prevalence adjusted and bias adjusted kappa (PABAK) and was near perfect (kappa mean 0.82 [range 0.66-1], PABAK 0.97 [range 0.94-1]) [43].

### Statistical Analysis

Means (SD) for each MARS subscale and the overall MARS score were calculated using Microsoft Excel 2016 for each app. A summary score was calculated for each app type (ie, recipe and recipe managers, meal planners, shopping lists, family organizers, and food choice apps) along with an overall mean score for all apps. The mean (SD) number of BCTs per app and app type was calculated, and the total number of apps from each app type incorporating the BCT was presented graphically. The presence of behavioral content and features and technical features was tallied for each app type and for all apps.

## Results

### App Selection

**Figure 1:**
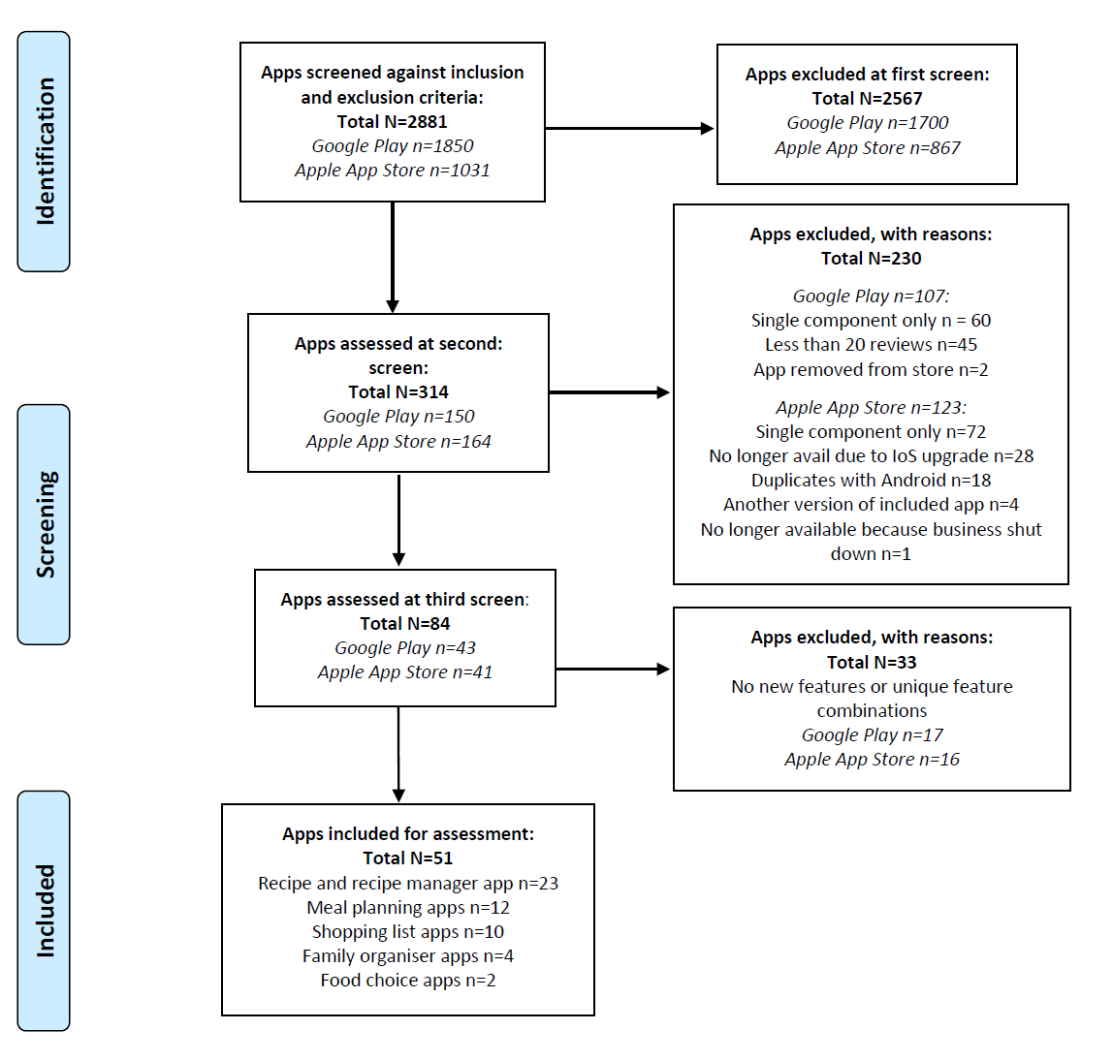
PRISMA (Preferred Reporting Items for Systematic Reviews and Meta-Analyses) flow diagram for popular, commercially available food and nutrition-related apps addressing parental food provision.

A total of 2881 apps were screened across the Google Play Store and Apple App Store. The final number included for assessment was 51 (see Figure 1).

### App Characteristics

Selected apps fell into 5 categories of app type: (1) recipe and recipe manager apps, which provided recipes or digital storage of recipes; (2) meal planning apps, which allowed the planning and recording of meals in advance; (3) shopping list apps, which allowed recording of grocery items for purchase; (4) family organizer apps, which included meal planners and shopping lists synced between family members; and (5) food choice apps, which provided nutrition or produce information to support food purchasing (see Multimedia Appendix 1 for app details and MARS scores).

Recipe and recipe manager apps were the most common app type in the sample (45%, 23/51), followed by meal planning apps (24%, 12/51). Almost all apps were developed by commercial enterprises, with the exception of 1 app developed by a government body and another by a nongovernment research institute in collaboration with a private health insurer. Approximately one-third (31%, 16/51) of apps were free to download and use (see Multimedia Appendix 2). The primary behavioral targets of the apps included food purchasing (90%, 46/51), meal preparation (76%, 39/51), meal planning (47%, 24/51), and food choice (10%, 5/51). Half (51%, 26/51) of the apps operated primarily on input from the app user, with shopping lists and family organizers being most reliant on user data input. Only one-quarter of apps incorporated both significant user data input along with app information output (25%, 13/51).

### App Quality

The mean MARS score for app quality was highest for food choice apps and family organizer apps (mean 3.5 [SD 0.6] out of 5 for each), followed by recipe and recipe manager apps (mean 3.4 [SD 0.5]). Shopping list apps had the lowest overall MARS scores, with half of the apps scoring below 2.5 (for MARS scores by app type, see Table 1, and by individual app, see Multimedia Appendix 1). Engagement was the lowest scoring domain for each app type, with shopping lists and meal planners performing the worst. Most app types scored well for functionality (mean across all app types 3.6 [SD 0.7]).

### App Content and Features

#### Behavioral Support Content and Features

App content and features relating to the provision of healthy family food are presented by app type (see Table 2, and for details by app, see Multimedia Appendix 2). Several common app features supported the use of key healthy food coping strategies, for example, meal planners, shopping lists, and social supports. Meal planners were the primary feature of all 12 meal planning apps and featured in around half of the overall sample (51%, 26/51). Shopping lists featured almost universally (86%, 44/51) and where incorporated into other app types (as opposed to a stand-alone shopping list app), they generally offered automated list generation. Similarly, almost all (94%, 48/51) apps included the ability to share app content by email and/or social media.

Recipes and recipe managers (the primary feature of recipe and recipe manager apps, n=23) were present in more than half of the overall sample (recipes 33/51, 65% and recipe managers 28/51, 55%). Food preparation skills instructions were uncommon (14%, 7/51) and included either text, image, or video-based instructions. Reminders and/or prompts were included in almost a third of apps (27%, 27/51). A small number of apps included general and produce-related nutrition information (16%, 8/51), whereas only 3 apps (6%) included the ability to purchase food for delivery.

**Table 1:** Mean (SD) Mobile App Rating Scale (MARS) subscores and overall scores by app type.

MARS domain subscores and overall score	Recipe and recipe manager apps (n=23), mean (SD)	Meal planning apps (n=12), mean (SD)	Shopping list apps (n=10), mean (SD)	Family organizer apps (n=4), mean (SD)	Food choice apps (n=2), mean (SD)	All apps (N=51), mean (SD)
Engagement	2.7 (0.6)	2.5 (0.8)	2.1 (0.4)	3.2 (0.7)	2.7 (1.3)	2.6 (0.7)
Functionality	3.8 (0.6)	3.8 (0.7)	3.0 (0.9)	3.7 (0.6)	4.4 (0.2)	3.6 (0.7)
Aesthetics	3.6 (0.8)	3.2 (1.0)	2.9 (0.9)	3.7 (0.9)	2.8 (0.2)	3.3 (0.9)
Information	3.4 (0.4)	3.2 (0.6)	2.9 (0.5)	3.6 (0.5)	4.0 (0.7)	3.3 (0.6)
Overall score	3.4 (0.5)	3.1 (0.7)	2.7 (0.6)	3.5 (0.6)	3.5 (0.6)	3.2 (0.6)

**Table 2:** App behavioral support content and features presented by app type and across all apps

Behavioral support content or feature	Recipe and recipe manager apps (n=23), n (%)	Meal planning apps (n=12), n (%)	Shopping list apps (n=10), n (%)	Family organizer apps (n=4), n (%)	Food choice apps (n=2), n (%)	All apps (N=51), n (%)
Meal planners and meal plans	10 (44)	12 (100)	2 (20)	2 (50)	0 (0)	26 (51)
Shopping list	20 (87)	9 (75)	10 (100)	4 (100)	1 (50)	44 (86)
Social community or connectivity^a^	10 (44)	4 (33)	0 (0)	0 (0)	0 (0)	14 (27)
Other social supports^b^	23 (100)	11 (92)	9 (90)	4 (100)	1 (50)	48 (94)
Recipes	19 (83)	6 (50)	4 (40)	3 (75)	1 (50)	33 (65)
Recipe managers	13 (57)	6 (50)	7 (70)	2 (50)	0 (0)	28 (55)
Pantry or fridge manager	1 (4)	1 (8)	5 (50)	0 (0)	0 (0)	7 (14)
Food preparation skills instructions	6 (26)	1 (8)	0 (0)	0 (0)	0 (0)	7 (14)
Reminders and prompts^c^	4 (17)	4 (33)	5 (50)	1 (25)	0 (0)	14 (27)
Encouragement and incentives^d^	8 (35)	1 (8)	4 (40)	2 (50)	0 (0)	15 (29)
Produce purchasing information	0 (0)	0 (0)	0 (0)	0 (0)	1 (50)	1 (2)
Produce storage information	1 (4)	0 (0)	0 (0)	0 (0)	1 (50)	2 (4)
Produce nutrition information	1 (4)	1 (8)	1 (10)	0 (0)	2 (100)	5 (10)
Recipe nutrition information	6 (26)	3 (25)	1 (10)	0 (0)	0 (0)	10 (20)
Other nutrition information	2 (9)	1 (8)	0 (0)	0 (0)	0 (0)	3 (6)
Food purchase and delivery	1 (4)	1 (8)	1 (10)	0 (0)	0 (0)	3 (6)

^a^Community (with following), upload recipes or images, rate, review, like, and comment.

^b^Sharing to social media, sending via email, shared calendar, and private texting.

^c^Recipe suggestions on entering the supermarket, supermarket proximity alert, and reminders (to cook, plan meals, and shop).

^d^Positive messages, points, rewards, competitions, sales or discounts, and other notifications (eg, new content and offers).

**Figure 2:**
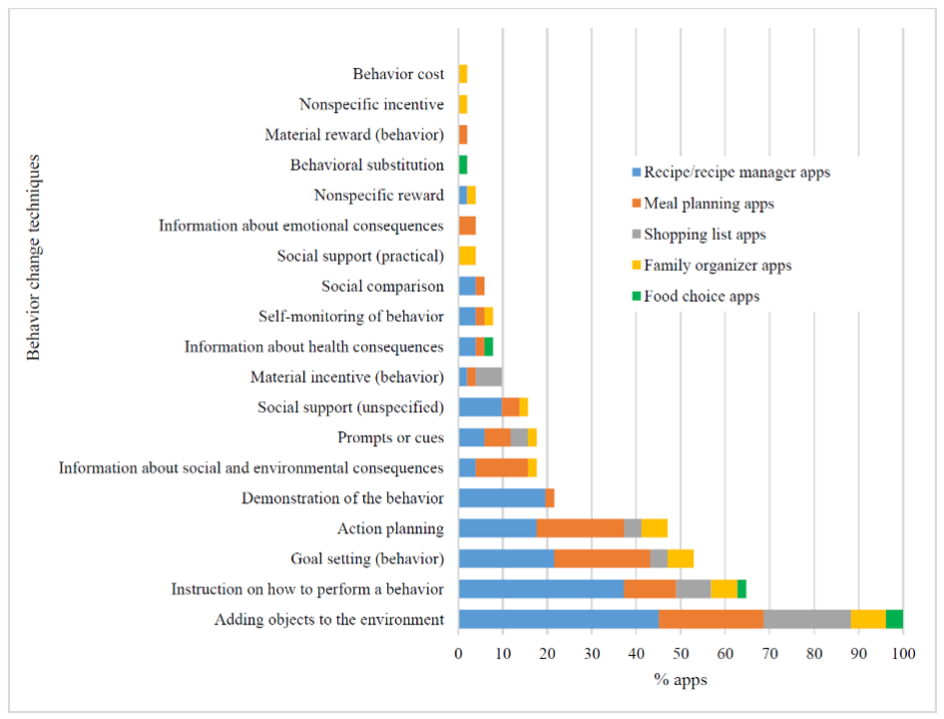
Proportion of apps identified with Behavior Change Technique present, by mobile app type.

Of 93 BCTs in the taxonomy, 19 were identified as being present across the 51 apps, with a mean (SD) of 3.9 (1.9) per app ranging from 1 to 10 (see Figure 2 and Multimedia Appendix 3). Family organizer apps followed by meal planning apps were identified as having the greatest number of BCTs (mean 5.5 [SD 3.1] and mean 4.8 [SD 1.9], respectively). Recipe and recipe manager apps included an average of 4 BCTs per app (mean 3.9 [SD 1.5]), whereas food choice apps and shopping list apps were identified as including the least number of BCTs (mean 2.5 [SD 0.7] and mean 2.3 [SD 0.8], respectively). The only BCT that was identified as being present across all apps was *Adding objects to the environment*. This was because of features such as shopping lists and meal planners that were thought to add objects to the environment that may subsequently enable a behavior relating to healthy food provision. Recipe and recipe managers commonly included the BCT *Instruction on how to perform the behavior* (83%, 19/23), owing to the inclusion of recipes with step-by-step instructions. Furthermore, 92% (11/12) and 83% (11/12) of meal planning apps included BCTs *Goal setting (behavior)* and *Action planning*, owing primarily to the ability to plan meals in advance.

#### Technical Features

Technical features were grouped separately as they were unlikely to directly support behavior but remained important to the overall functioning and engagement of the mobile apps (Table 3 and Multimedia Appendix 2). Two-thirds of apps (69%, 35/51) allowed some level of personalization, such as a customized recipe display based on food preferences, dietary requirements, or number of serves required. More than half of all apps (57%, 29/51), predominantly recipe and recipe manager apps, included practical features such as cooking timers, unit converters (ie, cups to milliliters), voice input of data, hands free commands, and automatic screen lock to prevent the device from sleeping while the app is in use. A little over half of the apps allowed syncing between devices and cloud backup (59%, 30/51 and 57%, 29/51, respectively).

**Table 3:** Technical features presented by app type and across all apps.

Technical feature	Recipe and recipe manager apps (n=23), n (%)	Meal planning apps (n=12), n (%)	Shopping list apps (n=10), n (%)	Family organizer apps (n=4), n (%)	Food choice apps (n=2), n (%)	All apps (N=51), n (%)
Personalization^a^	20 (87)	9 (75)	4 (40)	1 (25)	1 (50)	35 (69)
Practical features^b^	17 (74)	4 (33)	7 (70)	1 (25)	0 (0)	29 (57)
Syncing between devices	12 (52)	6 (26)	8 (80)	4 (100)	0 (0)	30 (59)
Cloud backup	14 (61)	5 (42)	7 (70)	3 (75)	0 (0)	29 (57)
User or family profile^c^	7 (30)	3 (25)	0 (0)	3 (75)	0 (0)	13 (25)
Miscellaneous and optional purchases^d^	4 (17)	2 (17)	5 (50)	2 (50)	0 (0)	13 (25)
Search and display options^e^	19 (83)	5 (22)	8 (80)	4 (100)	1 (50)	37 (73)
Other input options^f^	6 (26)	6 (26)	10 (100)	3 (75)	1 (50)	26 (51)
Requires log-in	12 (52)	6 (26)	7 (70)	3 (75)	0 (0)	28 (55)
Web access required	21 (91)	10 (83)	5 (50)	4 (100)	2 (100)	42 (82)

^a^Food preferences, dietary requirements, favorites lists, scale recipes to serves required, and add notes or rating to recipes (private).

^b^Prevents device from sleeping, voice command, audio reading, hands free, smart watch compatible, cooking timers, and unit conversions.

^c^Individual profile or profile of individual family members or family as a whole.

^d^To-do lists and optional purchases (eg, hard copy cookbook and cooking equipment).

^e^Search functions, for example, by ingredient, recipe name, and category (eg, vegetarian), and novel search functions, for example, by shaking device and by photo.

^f^Common items lists, history or recurring items, barcode scanners, add images, coupons, and loyalty cards.

## Discussion

### Principal Findings

This review identified and assessed commercially available food and nutrition-related mobile apps addressing family food provision. Most apps provided behavioral support for the use of healthy food coping strategies, although supports were biased toward planning behaviors, which may appeal to some but not all users. App features and content mapped to relatively few BCTs, with the higher quality family organizer apps, meal planning apps, and recipe and recipe manager apps incorporating the greatest number of techniques, respectively. Recipe and recipe manager apps, meal planning apps, and family organizers with integrated meal planning and shopping lists were found to be highly functional with regards to their performance and ease of use and incorporated a range of behavioral support features that could be used to address barriers to healthy food provision, such as time scarcity and cognitive load.

### App Characteristics and Quality

The majority of apps targeted meal planning and shopping list use, both considered healthy food coping strategies [13]. Although these food coping strategies are associated with healthier food preparation practices, they are best suited to those more inclined to plan [15]. Few apps effectively addressed food coping strategies such as preparing meals with few ingredients on hand, utilizing healthy convenience foods (ie, frozen or canned products and meal box kits), or seeking support. Furthermore, observed features often required extensive data input (eg, recipe managers and family organizers), which may be a barrier to app engagement or use [44].

Although most apps were generally functional in terms of their performance, ease of use, navigation, and gestural design, their low ratings for the engagement domain of the quality assessment was a concern, given this is a key predictor of long-term use [23]. A recent review of 11 weight loss apps addressing food-purchasing behavior reported similar findings [33], whereas, others have identified concerns regarding information quality and highlighted the need for evidence-based content [34]. However, as the information within the apps assessed in this review was mostly limited to recipes or food skills, the information quality rating is less relevant. The evidence base of such apps should be in their delivery of behavioral supports, to ensure that they have a positive influence on the food provision process.

### Behavioral Analysis

Mobile app behavioral supports such as shopping lists, meal planners, and recipe managers have the advantage of delivering BCTs in the real world, when behaviors are likely to occur, thus improving the chance of positively shaping behavior [21,22]. However, the number of BCTs identified in the present sample of apps was lower compared with similar reviews of weight loss and general nutrition apps [31,34], reflecting the development of these apps for commercial purposes rather than for behavior change or health promotion. This indicates significant scope for increasing the behavior change potential of future apps in this space.

There were a number of app types and features that should be considered in the development of future evidence-based, behavioral change theory–driven apps targeting food provision in families. Meal planning apps and features, supporting the formation of intentions to prepare a healthy meal, were identified as including the second largest mean number of BCTs. Most notably, they incorporated *Goal Setting (behavior)* and *Action Planning*. The 2 meal planning apps with the highest MARS scores and largest number of BCTs allowed the user to outsource some aspects of the planning and purchasing process. One included automated meal plans and shopping lists produced using an internal bank of recipes, whereas the other offered meal box kit ordering and delivery. These apps could be suitable for those not naturally inclined to plan and willing to relinquish some decision making regarding meals. However, inadequate personalization, complex recipes, and the high cost associated with ingredients and box kits may be barriers to the widespread use of such apps.

Shopping lists as a stand-alone app type generally failed to offer more than the conventional paper and pen method, so it was unsurprising that they performed poorly on all domains of the MARS and mapped against very few BCTs. Where shopping lists were incorporated into other app types and allowed automatic list generation through recipes, they have the potential to reduce the time burden associated with shopping list writing. Linking to Web-based grocery ordering would add a further efficiency; however, this feature was surprisingly uncommon, only being incorporated into 2 of 51 apps.

Another feature with the potential to increase efficiencies relating to food purchasing is the ability to sync grocery lists between family members (ie, a shared shopping list). This feature could be utilized to share the mental and physical load of planning and purchasing food. Family organizers generally offered the ability to share such tasks among family members but most were expensive (eg, up to Aus $69.99 per year subscription), requiring an ongoing subscription to access such features. Furthermore, they required significant data input and are likely suited to those with established planning skills.

Few apps incorporated timely reminders and prompts, which is a missed opportunity to take advantage of mobile apps ability to offer ecological momentary intervention [22]. If used appropriately (ie, not overwhelmingly) and timed to coincide with the performance of food-related behaviors, reminders and prompts in the form of push notifications could act to reduce the mental load of the food provision process. Supermarket proximity alerts and reminders of the planned evening meal were effective, albeit uncommon, examples of such push notifications, delivering the BCT *Prompts and cues.*

Most of the apps assessed provided limited information, generally in the form of recipes and food skills, which is consistent with the move toward more data input style apps. This content was associated with *Instruction on how to perform a behavior* and where video or image content was included, *Demonstration of the behavior*. However, most apps providing recipes or food skills were not focused on healthy food preparation or use of healthy food coping strategies (ie, utilizing frozen or canned foods, cooking from few ingredients), and few directly targeted families. Nutrition information delivered in the context of food purchasing, such as in 1 reviewed app that suggested healthier alternatives to scanned products, may be more likely to support behavior change than generic nutrition information. However, it is possible that the way information is presented and the functionality of the app delivering it determines its efficacy in changing behavior; For example, the convenience of the information (ie, barcode scanners for searching) and the pairing of recipes with relevant food skills videos, hands free commands, single directions displayed per page, and text to speech functions.

### Review Strengths and Limitations

Although the search strategy of this review was systematic and based on similar reviews of commercial apps for nutrition and weight management [31,32,34], it was limited by the lack of standard methodology for searching commercial mobile app stores. Lack of standardized search methods and limited and variable information provided in app descriptions made it difficult to ensure all eligible apps were captured, particularly high-quality apps. There were also limitations relating to the use and interpretation of the MARS score. The information quality domain was limited to assessing the accuracy of the app description and the credibility of the app developer in the absence of assessable information and should, therefore, be interpreted with caution. Moreover, although family organizer apps and food choice apps scored the highest MARS ratings, they were based on only 4 and 2 apps, respectively. Finally, the coding of BCTs was limited to features and content that could be accessed or viewed within the assessment period. Therefore, some push notifications may have been overlooked, whereas lengthy blogs within apps were excluded from detailed analysis.

Despite its limitations, this review assessed a large number of apps and provides unique information about their behavior change potential by not only describing and assessing app scope, characteristics, and quality but also through a behavioral analysis of app content and features. Reviewer training, along with the use of a second reviewer in a 20% sample, improves the objectivity and accuracy of the data extracted and assessed in this review. The present target group is families, but the findings have applications to food planning, purchasing, and preparation behaviors more generally.

### Implications for Practice and Future Research

The findings of this review suggest that recipe and recipe manager apps, family organizer apps, and meal planning apps should be explored as viable options for nutrition promotion interventions. Future apps should combine a range of behavioral support features such as meal planners, shopping lists, simple recipes, reminders and prompts, and food ordering to reduce the burden of the food provision process and maximize behavior change potential. Consideration of food coping strategies other than meal planning, or the incorporation of skills training, prompts, and encouragement to plan meals, would make these apps applicable to people less inclined to plan. Although particular attention should be paid to personalization features, they should also provide a level of automation that reduces the need for excessive data input. Finally, researchers and developers should be mindful of the needs of modern families and consider the engagement qualities of such apps to ensure their effectiveness and longevity.

### Conclusions

This review, assessing commercially available food and nutrition-related apps for family food provision, demonstrates that apps could be used to deliver behavioral support for healthy food coping strategies. Future apps should include a wider range of features and BCTs to promote engagement and improve the behavior change potential of such apps.

## Appendix

Multimedia Appendix 1 Included app details and Mobile App Rating Scale scores.

Multimedia Appendix 2 App content and features.

Multimedia Appendix 3 Behavior change technique presence within apps, according to the behavior change technique taxonomy version 1.

## Abbreviations

BCT: behavior change technique
BCTTv1: behavior change technique taxonomy version 1
ICC: intraclass correlation coefficient
MARS: Mobile App Rating Scale
PABAK: prevalence adjusted and bias adjusted kappa
