# Supplementary material for: Mobile Apps to Support Healthy Family Food Provision: Systematic Assessment of Popular, Commercially Available Apps
Source: JMIR Mhealth Uhealth. 2018 Dec 21;6(12):e11867. doi: 10.2196/11867 (PMC6320405; doi:10.2196/11867)
Supplement: Multimedia Appendix 1 [file mhealth_v6i12e11867_app1.pdf]

Multimedia Appendix 1. Included app details and Mobile App Rating Scale scores

App name (developer name)	Version[a]	Operating system[b]	App pricing structure (cost[c] in AUD)	Target behaviours: Meal planning	Food purchasing	Meal preparation	Food choice	MARS score (per MARS domain & overall) [d]: Engagement	Functionality	Aesthetics	Information quality	MEAN score
**Recipe and recipe manager apps**												
All recipes (Allrecipes.com, Inc)	6.5.2	I/A	Free		X	X		3.0	4.0	3.7	4.0	3.7
Big oven (BigOven.com)	5.6.17 - 5.7.23	I/A	Freemium (subscription)		X	X		3.0	3.8	3.7	3.5	3.5
Change4Life Smart Recipes (Public Health England)	3.0.3	I	Free	X	X	X	X	3.2	4.8	4.0	3.8	3.9
Cheftap (Mindframe Design, LLC)	4.0.0.415 - 4.0.0.424	I/A	Freemium (subscription)			X		2.6	3.3	3.3	3.5	3.2
Clean and Green Eating (Clean & Green Lifestyle)	2.0.2	I/A	Paid (4.49)		X	X		2.8	4.8	5.0	3.0	3.9
Cookbook recipes (Riafy Technologies)	11.16.26	A	Freemium (subscription)	X	X	X	X	2.4	3.0	3.3	3.0	2.9
Cookooz (Soft Venture)	1.2	I	Free	X		X		1.8	3.8	3.0	3.0	2.9
Copy me that (Copy me that)	1.0.0.2 - 3.0.1	I/A	Freemium (subscription)	X	X	X		3.2	3.0	2.7	3.5	3.1
Epicurious (Conde Nast Digital)	6.2.7	I	Free		X	X		3.6	4.3	4.7	3.5	4.0
Jamie Olivers Ultimate Recipes (Zolmo)	3.6.1	I	Paid (10.99)		X	X		3.0	4.3	4.3	3.5	3.8
Kitchen stories (Kitchen Stories)	7.2.1A	I/A	Free		X	X		3.6	4.5	5.0	3.5	4.2
My cookbook (Maadinfo Services)	5.0.34	I/A	Freemium (subscription)		X	X		3.2	3.8	3.7	4.0	3.7
My Recipe Book (Cross Forward Consulting, LLC)	3.3.4	I	Paid (0.99)		X	X		2.2	4.0	3.0	3.0	3.1
Nigella: The quick Collection (Random House)	1.9	I	Paid (5.99)		X	X		2.4	3.5	3.7	3.5	3.3
Paprika (Hindsight Labs LLC)	1.4.2	I/A	Paid (6.99)	X	X	X		2.2	3.5	3.0	3.5	3.1
Pepperplate (Pepperplate Inc.)	2.8	I/A	Free	X	X	X		1.8	3.8	2.3	3.0	2.7
Recipe book (Recipe Book)	6.0.3.4	A	Freemium (subscription)[e]		X	X		3.4	4.0	3.7	3.5	3.6
RecipeCloud (Jacob Hull)	3.1.6	I	Freemium (subscription)			X		2.4	4.0	4.0	3.0	3.4
Recipe keeper (Tudorspan)	3.13.3.0 - 3.15.2.0	I/A	Freemium (7.99)	X	X	X		2.6	4.0	4.0	4.0	3.7
VideoMeals (iCM Development Inc.)	2.2	I	Paid (4.49)		X	X		3.0	2.8	3.3	3.0	3.0
What's for dinner? (Snazzy Software, LLC)	2.1	I/A	Freemium (4.82)	X	X	X		2.0	2.8	1.7	2.5	2.2
What to cook? (Vitaly Kuz'menko)	2.1	I	Paid (1.49)	X	X	X		2.0	3.5	2.7	2.5	2.7
Yummly (Yummly)	1.9.5.3 - 1.9.6.4	I/A	Free		X	X		3.0	3.8	4.7	4.0	3.9
			MEAN (SD) MARS score for recipes and recipe managers:					2.7(0.6)	3.8(0.6)	3.6(0.8)	3.4(0.4)	3.4(0.5)
**Meal planning apps**												
Chef plan (Alberto Gasparin)	1.7.0	I/A	Paid (1.49)	X	X			1.6	4.0	3.0	2.0	2.7
Hello fresh (Hello Fresh)	2.18.2 - 2.25	I/A	Free[f]	X	X	X		3.4	4.3	4.7	4.0	4.1
Mealime (Mealime Meal Plans Inc)	1.6.6 - 1.7.5	I/A	Freemium (subscription)	X	X	X		4.0	4.0	4.7	4.0	4.2
Meal Planner Pal (Gregg Evans)	2.1	I	Freemium (1.49)	X	X			1.8	2.3	1.7	2.5	2.1
MealsUp (Benjamin Styles)	1.6.16	I	Freemium (2.99)	X	X	X		2.6	4.0	3.7	3.5	3.4
Menu Planner (InnovaDev, LLC)	4.05	I	Paid (4.49)	X	X	X	X	2.2	3.3	3.0	3.0	2.9
My family meal planner (My Family Meal Planner)	2.5	I/A	Paid (4.81)	X	X	X		1.6	4.0	2.3	3.0	2.7
PlanBuyCook (Appetising Ideas Pty Ltd)	2.3.4	I	Paid (5.99)	X	X	X		3.4	4.8	4.0	3.5	3.9
Plateful (Happy Accident Apps)	1.4.5 - 1.4.6	I/A	Free	X				1.6	4.0	2.7	2.5	2.7
Recipe calendar (Harmonic Soft)	2.10 - 2.20	I/A	Freemium (4.99)	X	X	X		3.0	3.5	3.3	3.5	3.3
Today's Parent Mealtime (Rogers Media)	1.2	I/A	Free	X	X	X		3.0	4.3	3.7	3.5	3.6
Week menu (Bjorn Karlsson)	2.3.6	I	Paid (5.99)	X		X		2.0	3.3	2.0	3.0	2.6
			MEAN (SD) MARS score for meal planners:					2.5(0.8)	3.8(0.7)	3.2(1.0)	3.2(0.6)	3.1(0.7)

				Target behaviours				MARS score (per MARS domain & overall) [d]				
App name (developer name)	Version[a]	Operating system[b]	App pricing structure (cost[c] in AUD)	Meal planning	Food purchasing	Meal preparation	Food choice	Engagement	Functionality	Aesthetics	Information quality	MEAN score
Shopping list apps												
AnyList (Purple Cover Inc)	5.8	I	Freemium (subscription)		X			3.0	4.3	3.7	3.5	3.6
Grocery king (Pocket Labs)	2.66 - 2.75f	I/A	Freemium (6.99)		X			2.2	2.8	3.7	3.0	2.9
Grocery List (Dmitry Polevoy)	6.4	I	Paid (1.99)		X			1.6	1.3	2.0	1.7	1.6
Grocery tracker (easicorp)	11.22 - 11.34	A	Freemium (6.42)	X	X			1.8	2.0	2.7	3.0	2.4
H-E-B (H-E-B)	2.4.0	I/A	Free		X	X		2.2	4.0	3.7	3.0	3.2
Lister (Lister Studios)	5.6.20	A	Free		X			2.4	4.0	3.7	3.0	3.3
Mighty shopping list (Mighty Pocket)	4.0.151	A	Freemium (3.99)	X	X			1.8	2.8	2.0	3.0	2.4
Out of milk (Out of milk)	8.3.0_816	I/A	Free		X	X		2.0	3.3	3.7	3.5	3.1
Scan2List (MidCentury M109edia Inc.)	10.6.702	I	Freemium (subscription)		X			2.0	2.8	2.3	2.5	2.4
Shopping List Ease (inMarket Media, LLC)	2.36	I	Freemium (2.99)		X			1.8	3.3	1.7	2.5	2.3
MEAN (SD) MARS score for shopping lists:								2.1(0.4)	3.0(0.9)	2.9(0.9)	2.9(0.5)	2.7(0.6)
Family organizer apps												
Cozi (Cozi Inc.)	9.2.5610 - 9.3.5757	I/A	Freemium (subscription)	X	X	X		3.2	4.0	3.3	4.0	3.6
Organizer To-Do (Yadahome.com LLC)	3.10	I	Freemium (14.99)[g]		X	X		2.4	2.8	2.67	3.0	2.7
OurHome (OurHome)	3.13.2	I/A	Free		X	X		4.0	4.0	4.7	4.0	4.2
Picniic (Picnic Labs Inc.)	1.10 - 1.16	I/A	Freemium (subscription)		X	X		3.0	4.0	4.0	3.5	3.6
MEAN (SD) MARS score for family organizers:								3.2(0.7)	3.7(0.6)	3.7(0.9)	3.6(0.5)	3.5(0.6)
Food choice apps												
FoodSwitch (The George Institute for Global Health)	2.1	I/A	Free		X		X	3.6	4.5	3.0	4.5	3.9
Perfect produce (SparkPeople)	1.0	A	Free		X	X	X	1.8	4.3	2.7	3.5	3.1
MEAN (SD) MARS score for food choice apps:								2.7(1.3)	4.4(0.2)	2.8(0.2)	4.0(0.7)	3.5(0.6)
OVERALL MEAN (SD) MARS ratings:								2.6(0.7)	3.6(0.7)	3.3(0.9)	3.3(0.6)	3.2(0.6)

a Ranges are included where various versions of apps may have been assessed over the course of the study
b A = available on Android, I = available on iOS
c Cost only included where a paid / premium version was purchased and assessed, and the cost was a one-off payment, as per inclusion criteria
d Range 1-5, 5 indicating a higher quality app
e Payment for ad removal only
f App free to use, with optional meal box kit purchases
g Option to pay by subscription
