# Supplementary material for: Mobile Apps to Support Healthy Family Food Provision: Systematic Assessment of Popular, Commercially Available Apps
Source: JMIR Mhealth Uhealth. 2018 Dec 21;6(12):e11867. doi: 10.2196/11867 (PMC6320405; doi:10.2196/11867)
Supplement: Multimedia Appendix 2 [file mhealth_v6i12e11867_app2.pdf]

Multimedia Appendix 2. App content and features

		BEHAVIORAL SUPPORT CONTENT AND FEATURES															TECHNICAL FEATURES										
App name	Primary data direction	Food preparation skills instructions	Food purchasing info	Recipe nutrition info	Produce storage info	Produce nutrition info	Other nutrition info	Recipes	Recipe managers	Food purchase & delivery	Shopping list	Pantry / fridge manager	Reminders & prompts	Social community / connectivitv	Other social supports	Meal planners & meal plans	Encouragement & incentives	User / family profile	Personalisation	Practical features	Miscellaneous & optional purchases	Search & display options	Other input options	Requires login	Web access required	Syncing between devices	Cloud back-up
**Recipe and recipe manager apps**																											
All recipes	Input & output	X		X				X			X(a)		X	X	X			X	X	X		X			X	X	X
Big oven	Input & output							X	X(a)		X(a)		X	X	X		X	X	X			X	X		X		X
Change4Life Smart Recipes	Output	X		X			X	X			X(a)				X	X	X		X			X			X[b]		
Cheftap	Input							X	X(a)						X				X	X		X		X[c]	X	X	X
Clean and Green Eating	Output			X				X			X(a)				X		X		X		X	X			X[b]		
Cookbook recipes	Input & output						X	X	X(a)		X(a)		X	X	X	X		X	X	X		X		X	X		
Cookooz	Input							X	X(m)						X				X				X	X	X[b]		X
Copy me that	Input & output							X	X(a)		X(a)			X	X	X		X		X	X	X		X	X	X	X
Epicurious	Output	X						X			X(a)		X		X		X		X	X	X	X		X	X		
Jamie Olivers Ultimate Recipes	Output	X						X			X(a)				X		X		X	X		X	X		X		
Kitchen stories	Output	X		X	X	X		X			X(a)			X	X	X	X		X	X				X	X		
My cookbook	Input								X(a)		X(a)			X	X				X	X		X	X	X	X		
My Recipe Book	Input							X	X(a)		X(a)				X				X	X		X		X[b]	X	X	X
Nigella: The quick Collection	Output	X						X			X(a)				X				X	X		X					
Paprika	Input										X(a)				X	X			X	X		X		X[b]	X	X	X
Pepperplate	Input								X(m[d])		X(a)				X	X			X	X		X	X	X	X[b]	X	X
Recipe book	Input & output			X				X	X(m)		X(a)			X	X		X	X	X	X		X			X	X	X
RecipeCloud	Input & output							X	X(a)					X	X		X	X	X	X		X		X	X	X	X
Recipe keeper	Input							X	X(a)		X(a)				X	X			X			X			X		
VideoMeals	Output							X			X(a)			X	X	X			X	X		X		X		X	X
What's for dinner?	Input							X	X(m)		X(a)				X	X				X					X[b]	X	X
What to cook?	Input								X(a[e])		X(a)	X(a)			X	X				X		X	X		X[b]	X	X
Yummly	Input & output			X				X		X	X(a)			X	X			X	X		X			X	X	X	X
TOTAL		6	0	6	1	1	2	19	13	1	20	1	4	10	23	10	8	7	20	17	4	19	6	12	21	12	14
**Meal planning apps**																											
Chef plan	Input										X(m)				X	X							X		X[b]		
Hello fresh	Output			X			X	X		X				X	X	X	X	X	X					X	X	X	
Mealime	Input & output							X			X(a)				X	X		X	X	X		X		X	X	X	

App name	Primary data direction	BEHAVIORAL SUPPORT CONTENT AND FEATURES																TECHNICAL FEATURES									
		Food preparation skills instructions	Food purchasing info	Recipe nutrition info	Produce storage info	Produce nutrition info	Other nutrition info	Recipes	Recipe managers	Food purchase & delivery	Shopping list	Pantry / fridge manager	Reminders & prompts	Social community / connectivity	Other social supports	Meal planners & meal plans	Encouragement & incentives	User / family profile	Personalisation	Practical features	Miscellaneous & optional purchases	Search & display options	Other input options	Requires login	Web access required	Syncing between devices	Cloud back-up
Meal Planner Pal	Input										X(a)		X		X	X					X		X		X[b]		
MealsUp	Input								X(m)		X(a)		X		X	X							X	X	X	X	X
Menu Planner	Input					X			X(a)		X(a)	X(m)		X	X	X			X	X		X	X	X	X[b]	X	X
My family meal planner	Output			X				X			X(a)				X	X			X								
PlanBuyCook	Input & output	X						X	X(m)		X(a)				X	X			X	X	X	X	X				X
Plateful	Input								X(m)				X			X			X						X		
Recipe calendar	Input & output			X				X			X(a)		X	X	X	X		X	X	X				X	X	X	X
Today's Parent Mealtime	Output							X	X(m)		X(a)			X	X	X			X			X		X	X		X
Week menu	Input								X(m)						X	X			X			X	X		X	X	X
	TOTAL	1	0	3	0	1	1	6	6	1	9	1	4	4	11	12	1	3	9	4	2	5	6	6	10	6	6
Shopping list apps																											
AnyList	Input							X	X(a)		X(a)		X		X				X	X		X	X	X	X	X	X
Grocery king	Input								X(m)		X(a)	X(a)	X		X					X		X	X	X	X[b]	X	X
Grocery List	Input					X					X(m)	X(m)										X	X				
Grocery tracker	Input										X(m)	X(m)			X	X						X	X			X	
H-E-B	Input & output			X				X		X[f]	X(a)				X		X		X	X	X	X	X	X	X	X	X
Lister	Input								X(m)		X(a)		X		X		X			X			X			X	X
Mighty shopping list	Input								X(m)		X(a)	X(a)			X	X			X	X	X		X	X			
Out of milk	Input & output							X	X(a[e])		X(a)	X(m)			X		X		X	X	X	X	X	X	X	X	X
Scan2List	Input							X	X(m)		X(m)		X		X				X		X	X	X	X[b]	X	X	X
Shopping List Ease	Input								X(m)		X(m)		X		X		X			X	X	X	X	X[b]		X	X
	TOTAL	0	0	1	0	1	0	4	7	1	10	5	5	0	9	2	4	0	4	7	5	8	10	7	5	8	7
Family organizer apps																											
Cozi	Input							X	X(a)		X(a)				X	X		X		X	X	X	X	X	X[b]	X	X
Organizer To-Do	Input							X			X(a)				X	X	X				X	X	X		X[b]	X	
OurHome	Input										X(a)		X		X		X	X				X	X	X	X	X	X
Picniic	Input & output							X	X(a[e])		X(a)				X			X	X			X		X	X[b]	X	X
	TOTAL	0	0	0	0	0	0	3	2	0	4	0	1	0	4	2	2	3	1	1	2	4	3	3	4	4	3
Food choice apps																											
FoodSwitch	Output					X					X(m)				X							X	X		X[b]		
Perfect produce	Output		X		X	X		X											X						X		
	TOTAL	0	1	0	1	2	0	1	0	0	1	0	0		1	0	0	0	1	0	0	1	1	0	2	0	0

(a) = autopopulated e.g. recipe content clipped from the web, ingredients from recipes sent to shopping list; (m) = manual data input by text, image, barcode
a No apps addressed automatic motivation
b Required for emailing, sharing, syncing and/or cloud back-up only
c Only allows up to 15 recipes without login, 100 with login
d Can be imported via online content but only via website (manual entry only in app)
e Recipes not imported – weblink saved and recipe viewed via internal browser
f Ordering can only occur via website, but is linked to groceries selected within the app
Reminders & prompts = Recipe suggestions on entering the supermarket, supermarket proximity alert, reminders (to cook, plan meals, shop)
Social community / connectivity = Community (with following), upload recipes/images, rate, review, like, comment
Other social supports = Sharing to social media, sending via email, shared calendar, private messaging
Encouragement & incentives = Positive messages, points, rewards, competitions, sales/discounts, other notifications (e.g. new content, offers)
User / family profile = Individual profile or profile of individual family members / family as a whole
Personalization = Food preferences, dietary requirements, favourites, try or make lists, scale recipes to serves required, add notes or rating to recipes (private)
Practical features = Prevents device from sleeping, voice command, audio reading, hands free, smart watch compatible, cooking timers, unit conversions
Miscellaneous & optional purchases = To-do lists, optional purchases (e.g. hard copy cookbook, cooking equipment)
Search & display options = Search functions e.g. by ingredient, recipe name, category, novel search functions e.g. by shaking device, by photo
Other input options = common items lists, history/recurring items, barcode scanners, add images, coupons, loyalty cards
