# Supplementary material for: Mobile Apps to Support Healthy Family Food Provision: Systematic Assessment of Popular, Commercially Available Apps
Source: JMIR Mhealth Uhealth. 2018 Dec 21;6(12):e11867. doi: 10.2196/11867 (PMC6320405; doi:10.2196/11867)
Supplement: Multimedia Appendix 3 [file mhealth_v6i12e11867_app3.pdf]

Multimedia Appendix 3. Behavior change technique presence within apps, according to the behavior change technique taxonomy version 1

App name	BEHAVIOR CHANGE TECHNIQUE<br>1.1 Goal setting (behav)	1.4 Action planning	2.3 Self-mon of behaviour	3.1 Social support (unspec)	3.2 Social support (prac)	4.1 Instr on how perform behav	5.1 Info about health cons	5.3 Info about soc & envir cons	5.6 Info about emot cons	6.1 Demo of the behaviour	6.2 Social comparison	7.1 Prompts/cues	8.2 Behav substitution	10.1 Material incentive behav	10.2 Material reward behav	10.3 Non-specific reward	10.6 Non-specific incentive	12.5 Add objects to the envir	14.1 Behaviour cost	No. of BCTs per app
Recipe and recipe manager apps																				
All recipes				X		X				X		X		X		X		X		7
Big oven	X			X		X												X		4
Change4Life Smart Recipes			X			X	X	X		X								X		6
Cheftap	X		X			X		X										X		5
Clean and Green Eating						X												X		2
Cookbook recipes	X	X				X	X			X		X						X		7
Cookooz	X	X				X												X		4
Copy me that	X	X		X		X												X		5
Epicurious						X				X	X	X						X		5
Jamie Olivers Ultimate Recipes						X				X								X		3
Kitchen stories						X				X	X							X		4
My cookbook																		X		1
My Recipe Book						X												X		2
Nigella: The quick Collection						X				X								X		3
Paprika	X	X																X		3
Pepperplate	X	X																X		3
Recipe book				X		X				X								X		4
RecipeCloud				X		X												X		3
Recipe keeper	X	X				X												X		4
VideoMeals	X	X				X				X								X		5
What's for dinner?	X	X				X												X		4
What to cook?	X	X																X		3
Yummly						X				X								X		3
										Mean (SD) no. of BCTs identified in recipe and recipe manager apps:										3.9 (1.5)
Meal planning apps																				
Chef plan	X	X						X										X		4
Hello fresh	X	X				X			X	X	X			X	X			X		9
Mealime	X		X	X		X		X	X									X		7
Meal Planner Pal	X	X										X						X		4
MealsUp	X	X						X				X						X		5
Menu Planner	X	X																X		3

	BEHAVIOR CHANGE TECHNIQUE																			
App name	1.1 Goal setting (behav)	1.4 Action planning	2.3 Self-mon of behaviour	3.1 Social support (unspec)	3.2 Social support (prac)	4.1 Instr on how perform behav	5.1 Info about health cons	5.3 Info about soc & envir cons	5.6 Info about emot cons	6.1 Demo of the behaviour	6.2 Social comparison	7.1 Prompts/cues	8.2 Behav substitution	10.1 Material incentive behav	10.2 Material reward behav	10.3 Non-specific reward	10.6 Non-specific incentive	12.5 Add objects to the envir	14.1 Behaviour cost	No. of BCTs per app
My family meal planner						X	X	X										X		4
PlanBuyCook	X	X				X												X		4
Plateful	X	X						X										X		4
Recipe calendar	X	X		X		X		X				X						X		7
Today's Parent Mealtime	X	X				X												X		4
Week menu	X	X																X		3
Mean (SD) no. of BCTs identified in meal planning apps:																				4.8 (1.9)
Shopping list apps																				
AnyList						X												X		2
Grocery king												X						X		2
Grocery List																		X		1
Grocery tracker	X	X																X		3
H-E-B						X								X				X		3
Lister																		X		1
Mighty shopping list	X	X																X		3
Out of milk						X								X				X		3
Scan2List						X												X		2
Shopping List Ease												X		X				X		3
Mean (SD) no. of BCTs identified in shopping list apps:																				2.3 (0.8)
Family organizer apps																				
Cozi	X	X			X	X												X		5
Organizer To-Do	X	X				X												X		4
OurHome	X	X	X	X				X				X				X	X	X	X	10
Picniic					X	X												X		3
Mean (SD) no. of BCTs identified in family organizer apps:																				5.5 (3.1)
Food choice apps																				
FoodSwitch													X					X		2
Perfect produce						X	X											X		3
No. of food choice apps incorporating BCT:																				2.5 (0.7)
TOTAL no. of apps incorporating BCT:	27	24	4	8	2	33	4	9	2	11	3	9	1	5	1	2	1	51	1	3.9 (1.9)
